# Trends and Opportunities in Health Economic Evaluations of Prosthetic Care Innovations

**DOI:** 10.33137/cpoj.v4i2.36364

**Published:** 2021-09-21

**Authors:** L Frossard

**Affiliations:** 1 YourResearchProject Pty Ltd, Brisbane, Australia.; 2 Griffith University, Gold Coast, Australia.; 3 University of the Sunshine Coast, Maroochydore, Australia.; 4 Queensland University of Technology, Brisbane, Australia.

**Keywords:** Artificial Limbs, Bionic Limbs, Bone-Anchored Prosthesis, Cost-Effectiveness, Cost-Utility, Health Economic Evaluation, Health Technology Assessment, Prosthesis, Socket-Suspended Prosthesis

## Abstract

Overcoming obstacles to prosthetic fittings requires frequent tryouts of sockets and components. Repetitions of interventions are upsetting for users and place substantial economic burden on healthcare systems. Encouraging prosthetic care innovations capable of alleviating clinical and financial shortcomings of socket-based solutions is essential. Nonetheless, evidence of socio-economic benefits of an innovation are required to facilitate access to markets. Unfortunately, complex decisions must be made when allocating resources toward the most relevant health economic evaluation (HEE) at a given stage of development of an innovation. This paper first, aimed to show the importance and challenges of HEEs of intervention facilitating prosthetic fittings. Next, the main trends in HEEs at various phases of product development and clinical acceptance of prosthetic care innovations were outlined. Then, opportunities for a basic framework of a preliminary cost-utility analysis (CUA) during the mid-stage of development of prosthetic care innovations were highlighted. To do this, fundamental and applied health economic literature and prosthetic-specific publications were reviewed to extract and analyse the trends in HEEs of new medical and prosthetic technologies, respectively. The findings show there is consensus around the weaknesses of full CUAs (e.g., lack of timeliness, resource-intensive) and strengths of preliminary CUAs (e.g., identify evidence gaps, educate design of full CUA, fast-track approval). However, several obstacles must be overcome before preliminary CUA of prosthetic care innovations will be routinely carried out. Disparities of methods and constructs of usual preliminary CUA are barriers that could be alleviated by a more standardized framework. The paper concludes by identifying that there are opportunities for the development of a basic framework of preliminary CUA of prosthetic care innovations. Ultimately, the collaborative design of a framework could simplify selection of the methods, standardise outcomes, ease comparisons between innovations and streamline pathways for adoption. This might facilitate access to economical solutions that could improve the life of individuals suffering from limb loss.

## INTRODUCTION

Alfred Nobel (1833–1896) said the following about innovation “*If I have a thousand ideas and only one turns out to be good, I am satisfied*.”

In healthcare, the difference between a “good” or a not so good innovation is made during health technology assessment (HTA) and/or health economic evaluation (HEE).^1^ As defined in **APPENDIX 1**, these evaluations aim at understanding what is the value for money of a treatment. Simply put, payers want to make sure they get a bang for their buck!

This is tough question because the answer is rarely black and white. Nonetheless, addressing any concerns with socio-economical value of an intervention is a prerequisite to warrant access to market. Great but unaffordable treatments have little prospect of being adopted by healthcare policymakers.

**Figure 1:**
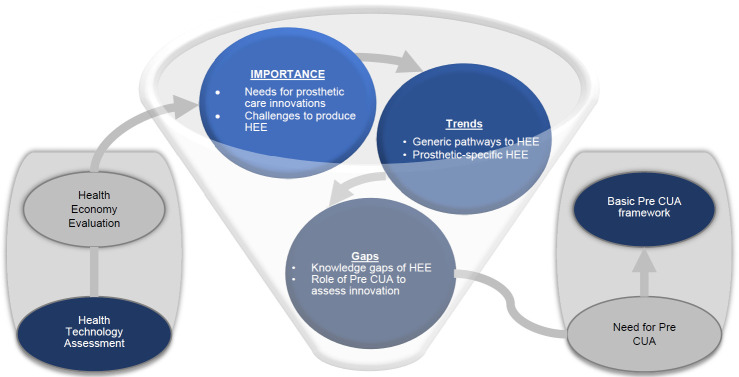
Overview of importance, trends and gaps of health economy evaluations (HEE) of prosthetic solutions leading to the need for basic framework of preliminary (Pre) cost-utility analysis (CUA) for prosthetic care innovations.

The paper deals with issues of health economic assessments specific to prosthetic care innovations as presented in **Figure 1**. First, the importance and challenges of HEEs of interventions facilitating prosthetic fittings are highlighted. Next, the main trends in HEEs of new healthcare technologies are outlined with particular emphasis on specific HEEs to consider during the course of development of innovations. Then, opportunities for a basic framework of preliminary assessments during the mid-stage of development of prosthetic care innovations are suggested. Finally, the paper concludes with some calls to action to further develop preliminary assessments.

## IMPORTANCE OF HEALTH TECHNOLOGY EVALUATIONS

This introductory section highlighted (A) the needs for solutions facilitating prosthetic fittings and (B) the current challenges to produce relevant health economic evaluations of prosthetic care innovations.

### Role of prosthetic care

Because the everyday ability of individuals suffering from limb loss to use an artificial limb is critical to their quality of life, clinical teams made bespoke recommendations intending to maximize comfort, stability and mobility of prosthetic fittings.^2,3^ Ultimately, this process incorporates all personalized interventions performed by a prosthetist around the choice and alignment of prosthetic components as well as the management of prosthetic attachment to the residuum including design, manufacture and adjustment of socket or osseointegrated implant.^4^

Outcomes of prosthetic fitting depends largely on the performance of prosthetic components.^5–11^ where Dillingham et al (2001) noted that 60% of amputees are satisfied with prosthetic characteristics such as weight, aesthetics and functionality (e.g. servicing, how easy the prosthesis is to use) and 57% of the traumatic lower limb amputees in the study expressed some dissatisfaction with prosthetic comfort.^12^ Since, studies showed that the use and satisfaction of prosthetic lower limb could be significantly improved when using advanced components such microprocessor-controlled knees compared to a non-microprocessor-controlled knees.^9,13,14^

Satisfactory prosthetic fitting might be compromised because of incongruous shapes of residuum (e.g., length, bulbous, volume change) and/or skin issues.^15–17^ Paterno et al (2018) and Meulenbelt et al (2009) report that that 63– 82% of lower limb amputees have problems with skin lesions.^2,18^ Turner and McGregor (2020) report that 48.0% of amputees and 65.7% of clinicians cited socket fit issues as the biggest factor impacting rehabilitation.^6^ And, sadly, Paterno et al (2018) and Meulenbelt et al (2009) found a 25–57% prosthetic abandonment rate and identified failed socket fit as a likely possible cause.^2,18^

### Demand for prosthetic care innovations

In many cases, overcoming obstacles to prosthetic fitting requires frequent tryouts of components and sockets fittings.^3^ Regular medical attention are, first and foremost, upsetting (e.g., pain), disruptive (e.g., sick leave) and costly (e.g., out-of-pocket expenses) for users.^19^ Repetitions of interventions also place a substantial economic burden on healthcare systems stressed to subsidize treatments beyond minimal prosthetic care standards.^20–22^ For example, the fitting of only a single socket per year might be approved by some healthcare organizations.^22^

Encouraging prosthetic care innovations that alleviate the clinical and financial shortcomings of current fitting options is essential (**APPENDIX 1**).^2,3,14,23–26^ Hence, efforts made by a bench of stakeholders (e.g., users, carers, clinicians, engineers, researchers, administrators) to develop and encourage new prosthetic care interventions to improve socket fittings and, eventually, eliminate socket attachments altogether (e.g., bone-anchored prostheses).^27–34^ These solution-finders will be called “promoters” of prosthetic care innovations throughout this paper and are shown in relationship to other concepts presented in this paper as a regrouping of individuals suffering from limb loss, providers of prosthetic solutions and administrators of healthcare organisations (**APPENDIX 1**) into a single collaborative group with common goals.^22,35–38^

Ultimately, prosthetic care innovations must be safe and efficient in ways that alleviate some adverse events (e.g., pain, slippage, pistoning, bell clapping, skin damages, falls), maximise functional outcomes (e.g., comfort, stability, mobility) and, preferably, enhance quality of life (e.g., Quality-Adjusted Life Year, Disability-Adjusted Life Year).^2,3,14,23–25,29,39,40^ Proofs of safety and efficacy of innovations are essential but no longer sufficient.^11^ Evidence of socio-economic benefits are also paramount.^4,30,37,38,41–47^

### Health economic evaluations of innovations

Ijzerman and Steuten (2011) identified that in order for societal benefits to be maximized three things must occur: 1) governments need more data on benefits arising when public resources are spent, 2) companies need more data to effectively manage their product development portfolios and 3) research programs at universities may need to be actively encouraged in this direction.^37^

Policymakers in healthcare organizations around the world adopt a reasoning more or less utilitarian when making decisions about medical care expenses.^37^ However, healthcare administrators are often obligated to confirm the value for money of interventions prior approval (e.g. fee-for-service, fee-for-value).^47–53^ For example, an HEE might be required to differentiate the four microprocessor-controlled knees assessed by Campbell et al (2020) all showing relative parity with regards to functional mobility, health state satisfaction and quality of life or injurious falls (i.e., C-Leg, Ottobock, Duderstadt, Germany; Orion, Blatchford Group, Hampshire, United Kingdom; Plie, Freedom Innovations, Irvine, California, United States; Rheo, Ossur, Reykjavik, Iceland).^14^ Recommendation for one knee or the other may be based on costs reduction of prosthetic care interventions.

The burden of HEE of an innovation also falls onto developers and manufacturers of technological solutions including attachments (e.g., liners, sockets, implants), artificial limb components (e.g., elbow, wrist, knee, ankle) and protective device (e.g., shock absorbers, failsafe).^38^ Steven et al (2019) suggested that solution developers must understand the value created by their interventions and act quickly on them to provide some forms of evidence of cost-effectiveness of their innovations.^48^ Failing to do so could seriously hinder access to market and adoption of their innovations. O'Malley (2010) indicated that the most common reason for the Australian Medical Services Advisory Committee to not recommend funding for new technology was not only insufficient clinical evidence but also the lack of proven cost-effectiveness presented during early stage of the examination process.^54^

### Making decisions about economic evaluations

Steven et al (2019) stated that HEE can be approached in a number of ways. They identified a range of approaches to compare the costs of health care services and possible cost savings which observe the consequences of an intervention and the effectiveness of that same intervention through a lens of outcomes that are valued patients, payers and providers, or which align with widely used global utility measures.^48^ They specified that the value of a prosthetic care intervention could be assessed using a range of cost-benefit, cost-consequence, cost-effectiveness and cost-utility analyses considering valuations of costs (e.g., monetary units) and a range of benefits. Ijzerman and Steuten (2011) specified that no single method will produce the right information for all decision makers. Each method has advantages and disadvantages and work for specific applications, as opposed to all applications.^37^ They suggested that a toolbox of methods must be used.

Unfortunately, the multitude of HEEs often leave promoters making challenging decisions around allocation of sufficient resources toward the most relevant HEE approach at a given point of an innovation development. Facilitating this decision-making process would start with an overview of the trends and specific ways HEEs can be done at various stages of development of an innovation.

## CURRENT TRENDS IN HEALTH TECHNOLOGY EVALUATIONS

This second section (A) reviewed generic pathways to assess health economic consequences of a new treatment at a given stage of product development and clinical acceptance and (B) highlighted selected studies that followed these pathways to assess prosthetic care interventions.

### Key concepts of health economic evaluations

As described in **APPENDIX 1**, HEE include, but not limited to, cost-effectiveness analyses (CEA) or cost-utility analyses (CUA). These terms are often used interchangeably although they are technically looking at different types of utilities. CEAs are concerned with a particular functional outcome of a treatment (e.g., walking speed). CUAs rely on self-reported quality of life status measured using standard surveys such as EQ-5D or 36-Item Short Form Survey (SF36). CUAs comparing usual and new treatments involve incremental cost-utility ratio (ICUR) based on incremental costs and utilities over time that could be compared to cost-effectiveness (CET) or, more often, willingness-to-pay (WTP) thresholds.^1,48,55^

Patient-centred assessments of global health-related quality of life might be influenced by prosthetic care to a certain extent. Therefore, these metrics might reflect only partially the benefits of a prosthetic intervention. However, outcomes of CUA reported in monetary units per quality-adjusted life-year (QALY) can be easily compared across other medical interventions or disease states. CUAs are commonly used to facilitate effective communication among healthcare professionals.^48,56,57^

### Health economic evaluations pathways

Promoters can be informed by an abundance of health economic research focusing on a broad range of fundamental and applied HEEs issues that could be more or less relevant (e.g., difference between pharmaceutical and medical technologies).^54,58^ Some studies provided valuable insights into ways outcomes of HEEs can facilitate the approval process of an innovation by a particular governmental healthcare system (e.g., Australian).^53,54,59–61^ Others explained the basic concepts of HEEs to clinicians and prosthetic care providers.^48,56,57^ Several landmark studies presented prosthetic-specific HEEs.^21,50–52,61–74^

Two studies were of particular interest because they can assist promoters to make an educated decision when choosing an HEE accordingly to the level of innovation development. Ijzerman and Steuten (2011) systematically described that early, preliminary and full CUAs can be conducted at the early, mid and late stage of clinical acceptance of any medical treatment, respectively.^37^ More recently, new insights were provided by Kannenberg and Seidinger (2019) who explained how these three types of CUAs should also be performed by prosthetic manufacturers at early, mid and late phase development of a prosthetic product.^38^ The authors indicated that CUA during the product's life cycle is beneficial in three ways. It allows potential cost-effectiveness to be estimated and included in investment decision processes and mitigates the risk of investing in technology unlikely to be cost-effective. It helps to prioritize between competing cost-effective concepts or technologies. It facilitates the identification of parameters having the largest impact on the likely cost-effectiveness of the product to be identified in order to best manage limited research funds.^38^

**Figure 2:**
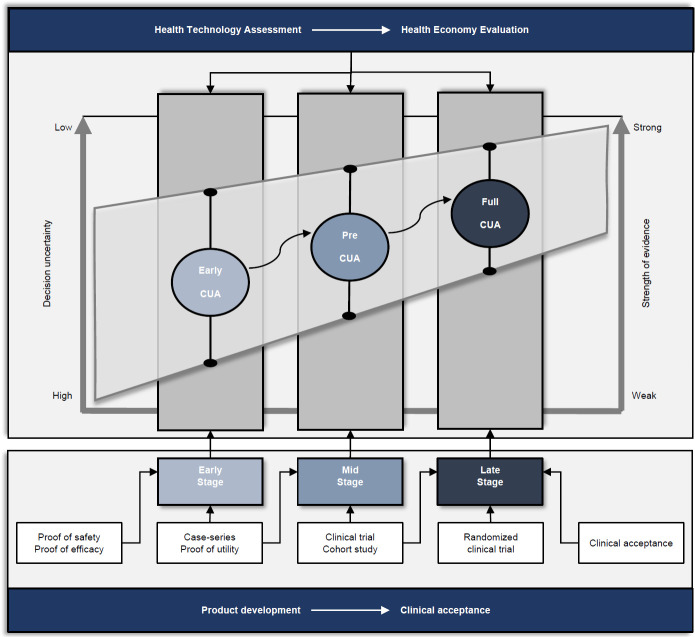
Overview of expected grading of decision uncertainty (i.e., high to low) and strength of evidence (i.e., weak to strong) of early, preliminary (Pre) and full cost-utility analysis (CUA) conducted during typical health technology assessments at early, mid and late phase of product development (manufacturer's perspective) and clinical acceptance (healthcare's perspective) of prosthetic care innovations, respectively.

**Figure 2** gives an overview synthesizing both approaches. Decision uncertainty and strength of evidence were suggested for early, preliminary and full CUAs during early, mid and late phase of product development (manufacturer's perspective) and clinical acceptance (healthcare's perspective) of prosthetic care innovations, respectively.

Next, the general principle, expected capacity to address Consolidated Health Economic Evaluation Reporting Standards (CHEERS) and Consensus Health Economic Criteria (CHEC) extended checklists, typical strengths and weaknesses as well as selected examples of prosthetic-focused CUAs.^75–77^ is briefly described. The decision was made to present the CUAs as they historically gained recognition starting from full, to preliminary and early CUAs rather than following the sequential timeline of their implementation. Appraisal of each type of CUAs using the CHEERS and CHEC-extended checklists were detailed in Supplementary material.

### Full cost-utility analyses

Traditionally, mainstream HEEs involved comprehensive or “full” CUAs essentially produced when innovations are gaining clinical acceptance after commercialisation. Full CUAs can be conducted from societal and/or healthcare perspectives. These CUAs usually rely on primary costs extracted from financial records expressed in monetary units as well as utilities measured by quality of life surveys expressed in QALY for cohorts of participants over an extended period of time (**APPENDIX 1**).^48,50–53,62,78,79^ Costs, utilities and ICURs are projected using Bayesian or Markov models based on plausible information extracted from primary studies for a series of scenarios over scalable time horizons (e.g., Years, decades, lifetime).^1,37,63,64,70,78,80,81^

It was postulated that conventional full CUAs should address strongly all items of the CHEERS and CHEC-extended checklists (**Table 1, Table 2**).

**Table 1:** Expected capacity (i.e., weak, moderate, strong) of typical early, preliminary (Pre) and full cost-utility analysis (CUA) to address the 27-item of the Consolidated Health Economic Evaluation Reporting Standards (CHEERS) checklist.

Section and item number		Recommendation	CUA		
			Early	Pre	Full
**Title and abstract**					
Title	1	Identify the study as an economic evaluation or use more specific terms such as “cost-effectiveness analysis”, and describe the interventions compared.	Strong	Strong	Strong
Abstract	2	Provide a structured summary of objectives, perspective, setting, methods (including study design and inputs), results (including base case and uncertainty analyses), and conclusions.	Strong	Strong	Strong
**Introduction**					
Background and objectives	3	Provide an explicit statement of the broader context for the study. Present the study question and its relevance for health policy or practice decisions.	Strong	Strong	Strong
**Methods**					
Target population and subgroups	4	Describe characteristics of the base case population and subgroups analysed, including why they were chosen.	Moderate	Moderate	Strong
Setting and location	5	State relevant aspects of the system(s) in which the decision(s) need(s) to be made.	Moderate	Strong	Strong
Study perspective	6	Describe the perspective of the study and relate this to the costs being evaluated.	Weak	Moderate	Strong
Comparators	7	Describe the interventions or strategies being compared and state why they were chosen.	Weak	Moderate	Strong
Time horizon	8	State the time horizon(s) over which costs and consequences are being evaluated and say why appropriate.	Moderate	Moderate	Strong
Discount rate	9	Report the choice of discount rate(s) used for costs and outcomes and say why appropriate.	Weak	Weak	Strong
Choice of health outcomes	10	Describe what outcomes were used as the measure(s) of benefit in the evaluation and their relevance for the type of analysis performed.	Weak	Weak	Strong
Measurement of effectiveness	11a	Single study-based estimates: Describe fully the design features of the single effectiveness study and why the single study was a sufficient source of clinical effectiveness data.	Weak	Weak	Strong
	11b	Synthesis-based estimates: Describe fully the methods used for identification of included studies and synthesis of clinical effectiveness data.	Weak	Weak	Strong
Measurement and valuation of preference based outcomes	12	If applicable, describe the population and methods used to elicit preferences for outcomes.	Weak	Weak	Strong
Estimating resources and costs	13a	Single study-based economic evaluation: Describe approaches used to estimate resource use associated with the alternative interventions. Describe primary or secondary research methods for valuing each resource item in terms of its unit cost. Describe any adjustments made to approximate to opportunity costs.	Weak	Weak	Strong
	13b	Model-based economic evaluation: Describe approaches and data sources used to estimate resource use associated with model health states. Describe primary or secondary research methods for valuing each resource item in terms of its unit cost. Describe any adjustments made to approximate to opportunity costs.	Weak	Weak	Strong
Currency, price date, and conversion	14	Report the dates of the estimated resource quantities and unit costs. Describe methods for adjusting estimated unit costs to the year of reported costs if necessary. Describe methods for converting costs into a common currency base and the exchange rate.	Strong	Strong	Strong
Choice of model	15	Describe and give reasons for the specific type of decision analytical model used. Providing a figure to show model structure is strongly recommended.	Moderate	Strong	Strong
Assumptions	16	Describe all structural or other assumptions underpinning the decision-analytical model.	Weak	Moderate	Strong
Analytical methods	17	Describe all analytical methods supporting the evaluation. This could include methods for dealing with skewed, missing, or censored data; extrapolation methods; methods for pooling data; approaches to validate or make adjustments (such as half cycle corrections) to a model; and methods for handling population heterogeneity and uncertainty.	Weak	Moderate	Strong
**Results**					
Study parameters	18	Report the values, ranges, references, and, if used, probability distributions for all parameters. Report reasons or sources for distributions used to represent uncertainty where appropriate. Providing a table to show the input values is strongly recommended.	Weak	Moderate	Strong
Incremental costs and outcomes	19	For each intervention, report mean values for the main categories of estimated costs and outcomes of interest, as well as mean differences between the comparator groups. If applicable, report incremental cost-effectiveness ratios.	Strong	Strong	Strong
Characterising uncertainty	20a	Single study-based economic evaluation: Describe the effects of sampling uncertainty for the estimated incremental cost and incremental effectiveness parameters, together with the impact of methodological assumptions (such as discount rate, study perspective).	Weak	Moderate	Strong
	20b	Model-based economic evaluation: Describe the effects on the results of uncertainty for all input parameters, and uncertainty related to the structure of the model and assumptions.	Weak	Weak	Strong
Characterising heterogeneity	21	If applicable, report differences in costs, outcomes, or cost effectiveness that can be explained by variations between subgroups of patients with different baseline characteristics or other observed variability in effects that are not reducible by more information.	Weak	Weak	Strong
**Discussion**					
Study findings, limitations, generalisability, and current knowledge	22	Summarise key study findings and describe how they support the conclusions reached. Discuss limitations and the generalisability of the findings and how the findings fit with current knowledge.	Strong	Strong	Strong
**Other**					
Source of funding	23	Describe how the study was funded and the role of the funder in the identification, design, conduct, and reporting of the analysis. Describe other non-monetary sources of support.	Strong	Strong	Strong
Conflicts of interest	24	Describe any potential for conflict of interest of study contributors in accordance with journal policy. In the absence of a journal policy, we recommend authors comply with International Committee of Medical Journal Editors recommendations.	Strong	Strong	Strong

**Table 2:** Expected capacity (i.e., yes, no) of typical early preliminary (Pre) and full cost-utility analysis (CUA) to address the 19-item Consensus Health Economic Criteria (CHEC) extended checklist.

Item	Questions	CUA		
		Early	Pre	Full
1	Is the study population clearly described?	Yes	Yes	Yes
2	Are competing alternatives clearly described?	Yes	Yes	Yes
3	Is a well-defined research question posed in answerable form?	Yes	Yes	Yes
4	Is the economic study design appropriate to the stated objective?	Yes	Yes	Yes
5	Is the chosen time horizon appropriate in order to include relevant costs and consequences?	No	No	Yes
6	Is the actual perspective chosen appropriate?	Yes	Yes	Yes
7	Are all important and relevant costs for each alternative identified?	No	No	Yes
8	Are all costs measured appropriately in physical units?	Yes	Yes	Yes
9	Are costs valued appropriately?	No	No	Yes
10	Are all important and relevant outcomes for each alternative identified?	No	No	Yes
11	Are all outcomes measured appropriately?	No	No	Yes
12	Are outcomes valued appropriately?	No	No	Yes
13	Is an incremental analysis of costs and outcomes of alternatives performed?	No	Yes	Yes
14	Are all future costs and outcomes discounted appropriately?	No	No	Yes
15	Are all important variables, whose values are uncertain, appropriately subjected to sensitivity analysis?	No	No	Yes
16	Do the conclusions follow from the data reported?	Yes	Yes	Yes
17	Does the study discuss the generalizability of the results to other settings and patient/client groups?	Yes	Yes	Yes
18	Does the article indicate that there is no potential conflict of interest of study researcher(s) and funder(s)?	Yes	Yes	Yes
19	Are ethical and distributional issues discussed appropriately?	Yes	Yes	Yes

Modelling CUAs can be comprehensive because of the breadth (e.g., scenarios) and depth (e.g., time horizon) of their analysis. Furthermore, uncertainty and sensibility of outcomes, shown by the size of the errors around the point estimates due to data sources (e.g., sample size) and/or to the process of evaluation (**APPENDIX 1**), tend to be well worked out and, possibly, relatively low compared to early and preliminary CUAs.^82^ Therefore, full CUA provide strong evidence supporting robust recommendations considered by decision makers (e.g., approval for funding).

However, modelling CUAs require substantial resources. Building models is labour intensive (e.g., determine scenarios, test assumptions). More importantly, Kannenberg and Seidinger (2019) noted the necessity of requiring the inclusion of outcome parameters, like health-related quality of life, in these models.^38^ This means that full CUAs produce their best outcomes when sufficient costs and utilities are known for large cohorts over an extended length of time in a given jurisdiction (e.g., within-trial and beyond-trial horizon studies).^83^ Evidence-based developments of new interventions takes time, particularly when the recommended clinical timelines are followed to demonstrate efficiency and safety (e.g., clinical trial registration, ethics approval, surgical learning curve, observation times, design of rehabilitation program). Several years might be needed to gather the costs and utilities required to complete primary and modelling CUAs. Consequently, mainstream CUAs can hardly inform promoters timely. Lack of timeliness is even more problematic with new prosthetic care technologies that are more susceptible to be superseded after five years.[60] Gallego et al (2011) described decisions to approve technology by committees and regulatory bodies, such as the Australian Medical Services Advisory Committee, typically occurs after the technology has evolved or is already commonly being used in practice.^60^ Ijzerman and Steuten (2011) also noted the problems with this approach, warning that many design decisions (e.g. target population, use setting, technology design features such as connectivity with data infrastructure, seamless integration with complementary technology, etc) are made in the early stages of product development and are difficult, expensive and/or impossible to change at a later date.^37^

Several studies used a full CUA to assess consequences of the provision of socket based solution including advanced prosthetic components such as microprocessor-controlled knees and energy storing and return feet as well as socket-free solutions including bone-anchored prostheses.^21,61,63–74^

### Preliminary cost-utility analyses

The issue of timeliness of full CUAs could be addressed by performing preliminary CUAs of innovations that could take place sometimes around the mid-stage of product development when clinical usage is still limited to small cohorts. Preliminary CUA is an option “in-between” early and full CUAs that considered innovations with a broad range of development status. Therefore, preliminary CUAs can be conducted using a wide spectrum of methods. They can involve primary data of actual (e.g., financial records) and/or simulated (e.g., purposely created schedules) costs expressed in monetary units as well as measured (e.g., quality of life surveys) and/or guesstimated (e.g., literature) utilities expressed in QALY for cohorts of participants over a somewhat lengthy time horizon.^48,50–52,62,78,79^

The assumption was made that typical preliminary CUAs have a weak and moderate capacity to address 9 (33%) and 8 (30%) of items in the CHEERS checklist, including 7 (44%) and 6 (38%) of items in the Methods as well as 2 (40%) and 2 (40%) of items in the Results sections, respectively (**Table 1**). It was estimated that preliminary CUAs should be capable to address 11 (58%) of items in the CHEC-extended checklists (**Table 2**).

Resources needed to conduct preliminary CUAs could varied depending on the sources of data considered. Estimating costs from schedules and utilities from literature might require less resources than extracting costs from financial systems and utilities from a survey for a cohort of convenient sample size. Preliminary CUAs can provide some indications of probable consequences of innovations. Practically, preliminary CUAs can generate primary information, in part or in whole, useful for modelling CUAs (e.g., costs and utilities estimates, scenario drafting).

However, preliminary CUAs are usually built around substantial assumptions based on best-estimates of costs and utilities at the time. Typical preliminary CUAs are characterised by narrow perspective, simple scenarios, and time horizons tentatively shorter than full CUAs. Further limitations are inherent to the mismatch of costs and utilities from incongruous jurisdictions, onsets and post-operative timelines. For example, actual costs extracted from an healthcare financial system over several years might be considered against estimated utilities based on studies performed in other countries measuring quality of life six months after the intervention.^50–52^ Finally, uncertainty and sensibility of preliminary CUAs might be only loosely considered and reported. Altogether, the weight of these limitations on the strength of evidence is less known weakening the recommendations. Unfavourable outcomes of preliminary CUAs might, at least, question and, possibly, stop further product commercialization and clinical considerations. A decision must be made whether favourable outcomes are deemed sufficient to pursue and eventually, readjust further developments.

Recent examples of preliminary CUAs of innovations looked at the benefits of transfemoral and transtibial bone-anchored prostheses from government prosthetic care perspective.^50–53^

### Early cost-utility analyses

Preliminary CUAs can provide timelier assessment than full CUAs. Nonetheless, there is a current trend in health economic literature arguing that preliminary CUAs are yet to provide sufficiently timely assessment of innovations. Hence, the promotion of early CUAs, also called “iterative economic evaluations” or “very early HTA” by Ijzerman and Steuten (2011), which pointed out that attempts have already been made, using “horizon scanning systems”, to include new, emerging technologies into health policy as it is developed. Other authors have referred to this as the use of “early warning systems”.^37^ Early CUAs tend to be constructed like preliminary CUAs but they rely more heavily on sparser costs and utilities data as well as sketchier assumptions. These analyses tend to be based on best guestimates of most likely costs and utilities collected with case-series studies and/or extracted from the literature often produced outside the relevant jurisdiction.

It was assumed that usual early CUAs have a weak capacity to address 15 (56%) of items in the CHEERS checklist including 11 (69%) of items in the Methods as well as 4 (80%) of items in the Results sections (**Table 1**). Preliminary CUAs might be incapable to address up to 9 (47%) of items in the CHEC-extended checklists (**Table 2**).

Early CUAs are affordable and timely. They could help to reduce or validate assumptions subsequently used in preliminary or modelling CUAs. Perhaps, the most valuable return on investment of early CUAs is to provide insight into the viability of the product and worthiness of the clinical introduction on an innovation, as described by Kannenberg and Seidinger (2019).^38^

As expected, outcomes of early CUAs are likely to have high uncertainty and sensibility leading to low level of evidence and only tentative recommendations. Early evidence of potential CUA might fast-track on-going innovation development. Limited prospects of CUA might raise questions about further allocation of resources to a product that has, ultimately, minimal chance to meet payer's expectations.

## GAPS IN EARLIER HEALTH ECONOMIC EVALUATIONS

This last section (A) presented the current consensus and knowledge gaps around earlier HEEs and (B) highlighted opportunities for developments of a basic framework of preliminary CUA.

### Benefits of earlier health economic evaluations

There is consensus around the weaknesses of full CUAs (e.g., lack of timeliness, resource-intensive) and strengths of early and preliminary CUAs, summarised in **Table 3**. Earlier CUAs have the potential to assist promoters to:

- Identify evidence gaps and headroom for improvements that generate insights into potential capacity of an innovation to alleviate the financial burden of prosthetic fittings.^37,50–52^
- Educate the design of primary and modelling studies including the planning (e.g., calculate statistical power, determine of sample size, obtain ethics approval), collection (e.g., mine data from financial records, design databases) and analysing (e.g., build model, draft scenarios, choose assumptions).^47^
- Fast-track approval from governing bodies like Australian Medical Services Advisory Committee. Gallego et al (2011) said that earlier CUAs can help to prioritize in which order new technologies are evaluated and allows for the fast-tracking of technologies which either have a least potential for harm or which have a great potential to benefit patients.^54,60^

**Table 3:** Typical strengths and weaknesses of the early, preliminary, and full cost-utility analyses (CUA) of prosthetic care innovations.

Strengths	Weaknesses
**Full CUA**	
Address strongly all 27 CHEERS items Capable to address all 19 CHEC items Comprehensive list of scenarios Scalable time horizon Strong understanding of uncertainty Strong understanding of sensibility High level of evidence Strong recommendations	Need of primary costs and utilities data Require substantial resources Lack of timeliness
**Preliminary CUA**	
Address strongly 37% of CHEER items Capable to address 58% of CHEC items Timeliness of information Identify evidence gaps Provide headroom for improvement Capable to generate primary data Educate design of full CUAs Fast-track approval	Address weakly 33% of CHEER items Uncapable to address 42% of CHEC items Variability of resources required Build around substantial assumptions Rely on best-known evidence Consider narrow perspective Consider plausible scenarios, Consider mid-term time horizon Mismatch costs and utilities data Limited understanding of uncertainty Limited understanding of sensitivity Moderate level of evidence Moderate strength of recommendations
**Early CUA**	
Address strongly 30% of CHEER items Capable to address 53% of CHEC items Require little resources Timeliness of information Early insights into product viability Early insights into clinical worthiness Identify evidence gaps Provide headroom for improvement Educate design of preliminary CUAs Facilitate fast-track approval	Address weakly 56% of CHEER items Uncapable to address 47% of CHEC items Build around substantial assumptions Rely on best-known evidence Consider narrow perspective Consider hypothetical scenarios, Consider short-term time horizon Rely of expected costs and utilities data Low understanding of uncertainty Low understanding of sensitivity Low level of evidence Low strength of recommendations

### Obstacles to earlier health economic evaluations

Ijzerman and Steuten (2011) pointed out that the emerging field of HTA research will likely gain prominence as it will help navigate the increasingly complex trade-offs that must be considered when making investments in medical product development and ensuring access to those products.^37^ However, earlier CUAs are far from being widely considered when developing new prosthetic solutions. Several obstacles must be overcome before earlier and, more particularly, preliminary CUAs of prosthetic care innovations would be routinely carried out by promoters.

One critical obstacle is the abundance of methods. Ijzerman and Steuten (2011) listed ten quantitative methods that could be used in earlier HTA (e.g., payback from research analysis, strategic business cases, health impact assessment, multi-criteria decision methods, choice-based preference methods, real options analysis, early health economic modelling, horizon scanning systems, clinical trial simulation, value-of-information analysis).^37^

Another obstacle is the multiple pathways for HEE relying on the same level of clinical evidence of utilities (**Figure 2**). Logically, early and full CUAs are indicated at early stage and after clinical acceptance, respectively. Initial clinical evidence provided by proof of utility and case-series could be used to perform an early and preliminary CUAs. Stronger evidence gathered during cohort study and clinical trial might be deemed sufficient to conduct a preliminary or full CUAs.

Disparities of methods and constructs of earlier CUAs (e.g., perspective, time horizon, discount, uncertainty, sensibility) have ripple effects limiting implementation of earlier CUAs. Cross-comparing outcomes of earlier CUAs between innovations might be challenging to interpret. Generalization of outcomes across healthcare organisations might be limited. Earlier CUAs might show a broad level of quality when appraised with standard CHEERS and CHEC-extended checklists, primarily designed for full CUAs (**Table 1, Table 2**). Altogether, disparity of outcomes also makes earlier CUAs scoring modestly in these checklists less likely to be published. The result of this is a sparsity of publications in prosthetic-focused scientific journals, let alone heath economics journals, the latter of which are inclined to consider that socio-economic research in prosthetics is for a niche audience. Literature review and meta-analyses of health economic evaluations failing to stratify publications accordingly to the three types of CUAs might appraise unfavourably the contribution of earlier CUAs.^84,85^ Therefore, this review might skew the perception on the overall quality of health economic evaluations of prosthetic care. Earlier CUAs might score less not because they are poorly done but because they are dealing with more unreliable datasets.

### Opportunities for basic framework of preliminary CUA

On a one side, every innovation is different. Each healthcare organisation has particular expectations. Promoters might choose a specific pathway for a given CUA depending on their confidence to make valid assumptions. Therefore, a preliminary CUA of an innovation could be unique.

One the other side, provision of prosthetic care follows a rather standardized process. Reimbursement are often made for categories of components (e.g., microprocessor-controlled knees.^14^ Prosthetists performed series of well-identified specific tasks related to prosthetic fitting (e.g., fitting of socket, choice of components, alignment of prosthesis), assessment of outcomes (e.g., comfort, stability, mobility) and reporting to payers (e.g., reimbursement claims).^47^ Indeed, each of these tasks is sufficiently codified to be individually supported by healthcare organisations (e.g., L-Codes). This means that most preliminary CUAs relying on estimated rather than primary costs could apply a template of schedule of allowable expenses. This typical matrix can present costs at the intersections of list of tasks in rows and timeline of interventions in columns (**APPENDIX 1**). Ideally, disruptive and economical innovations changing best prosthetic care practice should affect a schedule by reducing the price tag and/or the frequency of one or more tasks.

Furthermore, standard assessments are commonly used to quantify outcomes of prosthetic fittings using self-reported satisfaction (e.g., Orthotics and Prosthetics User's Survey, Quebec User Evaluation of Satisfaction with Assistive Technology, socket prosthetic comfort score), physical tasks (e.g., Berg Balance Scale, timed get-up and go, walking speed, 2-minute walk, 6-minute walk, functional ambulation profile, amputee mobility predictor with prosthesis) as well as specific (e.g., Questionnaire for Persons with a Transfemoral Amputation) and generic (e.g., EQ-5D, SF36) health-related quality of life with an innovation.**12,13,29,86**

Altogether, organisation of the delivery and assessment of prosthetic care might be sufficiently transferable across innovations to consider a more uniform approach to preliminary CUAs.^50–52^ This creates opportunities to explore the development of a basic framework including set constructs (e.g., perspective, time horizon, discount) and practical recommendations (e.g., funding cycles) specific to preliminary CUAs of the prosthetic care innovations (**APPENDIX 1**). This new approach to a preliminary CUA has the potential to simplify the selection of methods, standardise outcomes, ease comparisons between innovations and streamline pathways for adoption while facilitating the production of a body of literature on prosthetic health economics.

## CONCLUSION

This work showed that promoters must make complex decisions when attempting to establish the socio-economic values of prosthetic care innovations. It is commonly acknowledged that a unique type of CUA could not be applied at every stage of development of an innovation. Preliminary CUAs of innovations at the mid-stage of development is particularly valuable but challenging. Boundaries delineating preliminary CUAs from early and full CUA might be blurry pushing promoters to consider a wide range of methods.

The outcomes suggest that there are opportunities for collective design of a basic framework of a preliminary CUA of prosthetic care innovations. However, reaching consensus around a framework can be challenging because there is no formal forum capable to organise discussions outside of usual scientific peer-review channels. There is a need for an ad-hoc reference group involving promoters and heath economists specialized in prosthetics and medical aids. Ideally, this working group should be hosted by international (e.g., World Health Organisation Standards for Prosthetics and Orthotics Service Provision, International Society for Prosthetics and Orthotics) or national (e.g., American Orthotic and Prosthetic Association, Center for Orthotic and Prosthetic Learning and Outcomes/Evidence-Based Practice) governing bodies. Its missions could be to develop guidelines and, possibly, standards of HEEs of prosthetic care interventions including preliminary CUAs frameworks (e.g., set constructs, practical recommendations).

Ultimately, a wide adoption of a this collegial preliminary CUA framework will, hopefully, contribute to promote the routinely used preliminary CUA. It is anticipated that this framework should facilitate access to economical prosthetic care solutions improving the life of individuals suffering from limb loss worldwide.

## CALL TO ACTION

- Gather an ad-hoc reference group capable of (A) monitoring the current trends in HEEs of new healthcare technologies, (B) develop guidelines and, possibly, standards of HEEs of prosthetic care interventions, (C) promote the adoption of these guideline (e.g., publications of position papers, presentations at conferences).
- This working group could facilitate discussions between promoters of prosthetic care innovations around the use and validation of preliminary CUAs frameworks.
- Practically, these discussions should focus on the development of basic framework of a preliminary CUAs, more particularly set constructs and practical recommendations.

## DECLARATION OF CONFLICTING INTERESTS

The author is in the view that these competing interests do not conflict with the content of this manuscript. Laurent Frossard, Director and Chief Scientist Officer of YourResearchProject Pty Ltd, has worked as consultant for several organisations on non-related educational programs and projects of research focusing on recording loading data, developing of database to record clinical outcomes as well as drafting grants and manuscripts for Cognitive Institute, Exercise & Sports Science Australia, Griffith University, iPug Pty Ltd, Middlesex University, New Zealand Artificial Limb Service, Osseointegration Group of Australia Pty Ltd, OSSUR, Poly-Orthodox International, Queensland Artificial Limb Service, Queensland University of Technology, Return to Work-South Australia, South Australia Health, Tequir S.L, University of the New South Whales, University of the Sunshine Coast.

## SOURCES OF SUPPORT

This study was funded by YourResearchProject Pty Ltd.

## AUTHOR SCIENTIFIC BIOGRAPHY

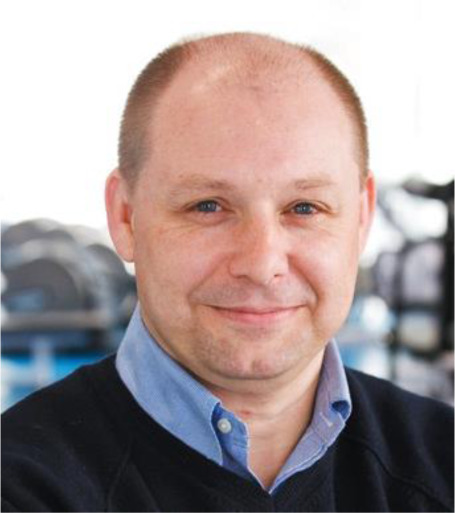



**Dr Laurent Frossard** is a bionic limbs scientist who is passionate about developing ground-breaking prosthetic solutions to improve the lives of individuals suffering from limb loss. He is internationally recognized as a researcher and an independent expert for his unique expertise in bionic limbs. He approaches bionic solutions from a holistic perspective, by integrating the prosthetic biomechanics, clinical benefits, service delivery, and health economics. Dr Frossard has over 25 years of experience, both in academia and in private industries in Australia, Canada, and Europe. He has collaborated with over 100 organizations worldwide. He is currently a Professor of bionics at the Griffith University, the Director and Chief Scientist Officer at YourResearchProject Pty Ltd, and Adjunct Professor at the Queensland University of Technology and the University of Sunshine Coast in Australia.

## LIST OF ABBREVIATIONS

CET: Cost-effectiveness threshold
CHEC: Consensus health economic criteria extended checklist
CHEER: Consolidated health economic evaluation reporting standards checklist
CUA: Cost-utility analysis
HEE: Health economic evaluation
HTA: Health technology assessment
ICER: Incremental cost-effectiveness ratio
ICUR: Incremental cost-utility ratio
QALY: Quality-adjusted life-year
SF36: 36-Item Short Form Survey
WTP: Willingness-to-pay threshold

## APPENDIX 1:

### Definition of key terms

**Table:**

**Basic framework of preliminary cost-utility analysis**	Generic canvas of preliminary cost-utility analysis including set constructs specific to prosthetic care innovations
**Cost-effectiveness analysis**	Form of economic analysis that compares the relative costs expressed in monetary value and particular functional outcome of a treatment (e.g., walking speed)
**Cost-utility analysis**	Form of economic analysis that compares the relative costs expressed in monetary value and health effects of various interventions converted into utilities expressed quality-adjusted life-year
**Health economic evaluation**	Comparative assessment of costs and outcomes of alternative health care technologies or health strategies providing incremental cost-outcome ratio, the relation of the estimated additional costs and the estimated additional outcome saved or lost by using an alternative health care technology
**Health technology assessment**	Systematic evaluation of the properties and effects of a health technology, addressing the direct and intended effects of this technology, as well as its indirect and unintended consequences, and aimed mainly at informing decision making regarding health technologies
**Modelling cost-utility analysis**	Form of analysis projecting of cost-utility based on decision-analytic models involving Bayesian or Markov models generally using plausible information extracted from primary studies
**Primary cost-utility analysis**	Form of analysis relying on actual costs extracted from financial records expressed in monetary units or actual utilities measured by quality of life surveys converted into quality-adjusted life-year
**Promoters of prosthetic care interventions**	Groups developing and encouraging prosthetic care interventions including individuals suffering from limb loss (users' perspectives), providers of prosthetic solutions (manufacturers' perspective), rehabilitation and prosthetic specialists (clinicians' perspective) and administrators of healthcare organisations (taxpayers perspective)
**Prosthetic care innovation**	New intervention susceptible to alleviate clinical shortcomings and financial burden of current prosthetic fitting options
**Schedule of allowable expenses**	Matrix of costs (monetary units of talk) at the intersection of rows corresponding to lists of tasks (type of expenses) and columns corresponding to onsets of tasks (time of expenses)
**Uncertainty and sensibility of health economic evaluations**	The size of the errors around the estimates of costs and utilities due to data sources (e.g., sample size) and/or to the process of evaluation (Markov modelling),

## References

[1] Tan-Torres Edejer T, Baltussen R, Adam T, Hutubessy R, Acharya A, Evans DB, et al. WHO guide to cost-effectiveness analysis [Internet]. World Health organisation. 2003; [Cited 2021, June 9]. Available from: https://www.who.int/choice/publications/p_2003_generalised_cea.pdf?ua=1DFID

[2] Paternò L, Ibrahimi M, Gruppioni E, Menciassi A, Ricotti L. Sockets for Limb Prostheses: A Review of Existing Technologies and Open Challenges. IEEE Trans Biomed Eng. 2018; 65(9):1996–2010. DOI: 10.1109/tbme.2017.2775100 29993506

[3] Klenow TD, Kahle JT, Fedel FJ, Ropp J, Highsmith MJ. Comparative Efficacy of Transfemoral Prosthetic Interfaces. J Prosthet Orthot. 2017; 29(3):130–136. DOI: 10.1097/jpo.0000000000000135

[4] Frossard L, Laux S, Lee Gow D, Berg D. Role of the prosthetist in provision of bone-anchored prostheses: governmental and practitioner perspectives. The AOPA Review. 2018; 3(1):26–27. https://eprints.qut.edu.au/123164/

[5] Raschke SU, Orendurff MS, Mattie JL, Kenyon DE, Jones OY, Moe D, et al. Biomechanical characteristics, patient preference and activity level with different prosthetic feet: a randomized double blind trial with laboratory and community testing. J Biomech. 2015; 48(1):146–52. DOI: 10.1016/j.jbiomech.2014.10.002 25480541

[6] Turner S, McGregor AH. Perceived effect of socket fit on major lower limb prosthetic rehabilitation: A clinician and amputee perspective. Archives of Rehabilitation Research and Clinical Translation. 2020; 2(3):100059. DOI: 10.1016/j.arrct.2020.100059 33543086 PMC7853327

[7] Collins DM, Karmarkar A, Relich R, Pasquina PF, Cooper RA. Review of research on prosthetic devices for lower extremity amputation. Crit Rev Biomed Eng. 2006; 34(5):379–438. DOI: 10.1615/critrevbiomedeng.v34.i5.20 17206920

[8] Van der Linde H, Hofstad CJ, Geurts AC, Postema K, Geertzen JH, van Limbeek J. A systematic literature review of the effect of different prosthetic components on human functioning with a lower-limb prosthesis. J Rehabil Res Dev. 2004; 41(4):555–70. DOI: 10.1682/jrrd.2003.06.0102 15558384

[9] Samuelsson KA, Töytäri O, Salminen AL, Brandt Å. Effects of lower limb prosthesis on activity, participation, and quality of life: a systematic review. Prosthet Orthot Int, 2012. 36(2): 145–58. DOI: 10.1177/0309364611432794 22307861

[10] Webster JB, Hakimi KN, Williams RM, Turner AP, Norvell DC, Czerniecki JM. Prosthetic fitting, use, and satisfaction following lower-limb amputation: a prospective study. J Rehabil Res Dev. 2012; 49(10):1493–504. DOI: 10.1682/jrrd.2012.01.0001 23516053

[11] Ghillebert J, De Bock S, Flynn L, Geeroms J, Tassignon B, Roelands B, et al. Guidelines and recommendations to investigate the efficacy of a lower-limb prosthetic device: a systematic review. IEEE Trans. Med. Robot. Bionics. 2019; 1(4):279–296. DOI: 10.1109/tmrb.2019.2949855

[12] Dillingham TR, Pezzin LE, MacKenzie EJ, Burgess AR. Use and satisfaction with prosthetic devices among persons with trauma-related amputations: a long-term outcome study. Am J Phys Med Rehabil. 2001; 80(8):563–71. DOI: 10.1097/00002060-200108000-00003 11475475

[13] Pezzin LE, Dillingham TR, MacKenzie EJ, Ephraim P, Rossbach P. Use and satisfaction with prosthetic limb devices and related services. Arch Phys Med Rehabil. 2004; 85(5):723–9. DOI: 10.1016/j.apmr.2003.06.002 15129395

[14] Campbell JH, Stevens PM, Wurdeman SR. OASIS 1: Retrospective analysis of four different microprocessor knee types. RATE. 2020;1–10. DOI: 10.1177/2055668320968476 PMC7649908 33224520

[15] Bui KM, Raugi GJ, Nguyen VQ, Reiber GE. Skin problems in individuals with lower-limb loss: literature review and proposed classification system. J Rehabil Res Dev. 2009; 46(9):1085–90. DOI: 10.1682/jrrd.2009.04.0052 20437314

[16] Kahle JT, Klenow TD, Highsmith MJ. Comparative effectiveness of an adjustable transfemoral prosthetic interface accommodating volume fluctuation: case study. Technology and Innovation. 2016; 18(2-3):175–183. DOI: 10.21300/18.2-3.2016.175 28066526 PMC5218538

[17] Gholizadeh H, Abu Osman NA, Eshraghi A, Arifin N, Chung TY. A comparison of pressure distributions between two types of sockets in a bulbous stump. Prosthet Orthot Int. 2016; 40(4):509–16. DOI: 10.1177/0309364614564022 25583929

[18] Meulenbelt HE, Geertzen JH, Jonkman MF, Dijkstra PU. Determinants of skin problems of the stump in lower-limb amputees. Arch Phys Med Rehabil. 2009; 90(1):74–81. DOI: 10.1016/j.apmr.2008.07.015 19154832

[19] Schaffalitzky E, Gallagher P, Maclachlan M, Ryall N. Understanding the benefits of prosthetic prescription: exploring the experiences of practitioners and lower limb prosthetic users. Disabil Rehabil. 2011; 33(15-16):1314–23. DOI: 10.3109/09638288.2010.529234 21050130

[20] Jordan RW, Marks A, Higman D. The cost of major lower limb amputation: a 12-year experience. Prosthet Orthot Int. 2012; 36(4):430–4. DOI: 10.1177/0309364612441489 22440579

[21] Boone DA. The economic value of mobility with a prosthesis. J Prosthet Orthot. 2019; 31(1S). DOI: 10.1097/JPO.0000000000000231

[22] Fish D. The Development of Coverage Policy for Lower Extremity Prosthetics: The Influence of the Payer on Prosthetic Prescription. J Prosthet Orthot. 2006; 18(6):125–129. DOI:10.1097/00008526-200601001-00017

[23] Papaioannou G, Mitrogiannis C, Nianios G, Fiedler G. Assessment of amputee socket–stump–residual bone kinematics during strenuous activities using Dynamic Roentgen Stereogrammetric Analysis. J Biomech. 2010; 43(5):871–878. DOI: 10.1016/j.jbiomech.2009.11.013 20047746

[24] Zheng YP, Mak AF, Leung AK. State-of-the-art methods for geometric and biomechanical assessments of residual limbs: a review. J Rehabil Res Dev. 2001; 38(5):487–504. 11732827

[25] Gholizadeh H, Osman NA, Eshraghi A, Ali S. Transfemoral prosthesis suspension systems: a systematic review of the literature. Am J Phys Med Rehabil. 2014; 93(9):809–23. DOI: 10.1097/PHM.0000000000000094 24743451

[26] Gerzina C, Potter E, Haleem AM, Dabash S. The future of the amputees with osseointegration: A systematic review of literature. J Clin Orthop Trauma. 2020; 11(Suppl 1): S142–S148. DOI: 10.1016/j.jcot.2019.05.025 31992935 PMC6977164

[27] Potter BK. From bench to bedside: we can (still) do better-moving towards more thoughtful, "constructive" amputations. Clin Orthop Relat Res. 2019; 477(8):1793–1795. DOI: 10.1097/CORR.0000000000000872 31335599 PMC7000012

[28] Overmann AL, Forsberg JA. The state of the art of osseointegration for limb prosthesis. Biomed Eng Lett. 2019; DOI: 10.1007/s13534-019-00133-9 PMC7046912 32175127

[29] Frossard L, Hagberg K, Häggström E, Gow DL, Brånemark R, Pearcy M. Functional outcome of transfemoral amputees fitted with an osseointegrated fixation: Temporal gait characteristics. J Prosthet Orthot. 2010; 22(1):11–20. DOI: 10.1097/JPO.0b013e3181ccc53d

[30] Frossard L, Ferrada L, Berg D. Survey data on the quality of life of consumers fitted with osseointegrated fixation and bone-anchored limb prostheses provided by government organization. Data in Brief. 2019; 26:104536. DOI: 10.1016/j.dib.2019.104536 31667297 PMC6811965

[31] Helgason B, Pálsson H, Rúnarsson TP, Frossard L, Viceconti M. Risk of failure during gait for direct skeletal attachment of a femoral prosthesis: a finite element study. Med Eng Phys. 2009; 31(5):595–600. DOI: 10.1016/j.medengphy.2008.11.015 19150253

[32] Frossard L, Haggstrom E, Hagberg K, Branemark R. Load applied on a bone-anchored transfemoral prosthesis: characterisation of prosthetic components – A pilot study. J Rehabil Res Dev. 2013; 50(5):619–634. DOI: 10.1682/JRRD.2012.04.0062 24013910

[33] Frossard L, Leech B, Pitkin M. Automated characterization of anthropomorphicity of prosthetic feet fitted to bone-anchored transtibial prosthesis. IEEE Trans Biomed Eng. 2019; 66(12):3402–3410. DOI: 10.1109/TBME.2019.2904713 PMC6926161 30872221

[34] Frossard L, Leech B, Pitkin M. Loading applied on osseointegrated implant by transtibial bone-anchored prostheses during daily activities: Preliminary characterization of prosthetic feet. J Prosthet Orthot. 2020; 32(4):258–271. DOI: 10.1097/jpo.0000000000000280 33013144 PMC7526518

[35] Highsmith MJ, Kahle JT, Lewandowski A, Klenow TD, Orriola JJ, Miro RM, et al. Economic evaluations of interventions for transtibial amputees: A scoping review of comparative studies. Technol Innov. 2016;18(2-3):85–98. DOI:10.21300/18.2-3.2016.85 28066519 PMC5218541

[36] Tai BB, Bae YH, Le QA. A systematic review of health economic evaluation studies using the patient's perspective. Value Health. 2016; 19(6):903–908. DOI: 10.1016/j.jval.2016.05.010 27712720

[37] Ijzerman MJ, Steuten LM. Early assessment of medical technologies to inform product development and market access: a review of methods and applications. Appl Health Econ Health Policy. 2011; 9(5):331–47. DOI: 10.2165/11593380-000000000-00000 21875163

[38] Kannenberg A, Seidinger S. Health economics: the perspective of a prosthetic manufacturer. J Prosthet Orthot. 2019; 31(1S). DOI:10.1097/JPO.0000000000000234 PMC10443514 37615010

[39] Eshraghi A, Osman NA, Gholizadeh H, Karimi M, Ali S. Pistoning assessment in lower limb prosthetic sockets. Prosthet Orthot Int. 2012; 36(1):15–24. DOI: 10.1177/0309364611431625 22269941

[40] Frossard LA, Tranberg R, Haggstrom E, Pearcy M, Brånemark R. Load on osseointegrated fixation of a transfemoral amputee during a fall: loading, descent, impact and recovery analysis. Prosthet Orthot Int. 2010; 34(1):85–97. DOI: 10.3109/03093640903585024 20196690

[41] Osseointegrated prosthetic implants for people with lower-limb amputation: a health technology assessment [Internet]. Ont Health Technol Assess Ser. 2019;[Cited 2021, June 9]. Available from: http://www.hqontario.ca/evidence-to-improve-care/journalontario-health-technology-assessment-series PMC6939984 31911825

[42] Martin R. Rapid review of osseointegration/direct skeletal fixation-A report for NHS England [Internet]. Bazian Ltd: UK, 2016; [Cited 2021, June 9]. Available from: https://www.ispo.org.uk/resources/Bazian-Report.pdf

[43] Amsan AN, Nasution AK, Riau P, Ramlee MH. A short review on the cost, design, materials and challenges of the prosthetics leg development and usage. International Conference of CELSciTech 2019-Science and Technology track (ICCELST-ST 2019) 2019; 59–64. DOI:10.2991/iccelst-st-19.2019.12

[44] Kaulback K, Jones A. Osseointegrated prosthetic implants for lower limb amputation: A review of clinical effectiveness, cost-effectiveness and guidelines [Internet]. Ottawa (ON): Canadian Agency for Drugs and Technologies in Health; 2017; [Cited 2021, June 9]. Available from: https://pubmed.ncbi.nlm.nih.gov/28825780/28825780

[45] Frossard L, Ferrada L, Berg D. Survey on the quality of life of consumers fitted with osseointegrated fixation and bone-anchored limb prostheses provided by government organization. 2019; Mendeley data. DOI: 10.17632/bkbxxmrhfh.1 PMC6811965 31667297

[46] Frossard L, Ferrada L, Quincey T, Burkett B, Berg D. Development of a Government Continuous Quality Improvement Procedure for Assessing the Provision of Bone Anchored Limb Prosthesis: A Process Re-Design Descriptive Study. Can Prosthet Orthot J. 2018; 1(2). DOI: 10.33137/cpoj.v1i2.31326

[47] Frossard L, Merlo G, Quincey T, Burkett B, Berg D. Development of a procedure for the government provision of bone-anchored prosthesis using osseointegration in Australia. PharmacoEconomics. 2017; 1(4):301–314. DOI: 10.1007/s41669-017-0032-5 PMC5711750 29441506

[48] Stevens PM, Highsmith MJ, Sutton B. Measuring value in the provision of lower-limb prostheses. J Prosthet Orthot. 2019; 31(1S). DOI: 10.1097/JPO.0000000000000232

[49] Frossard L, Debra B. Australian innovations of health services and economic evaluation of bone-anchored prosthesis using osseointegration, in Australian Orthotic Prosthetic Association (AOPA) Congress. 2017; Melbourne, Australia.

[50] Frossard L, Ferrada L, Quincey T, Berg D. Cost-Effectiveness of Transtibial Bone-Anchored Prostheses Using Osseointegrated Fixation. J Prosthet Orthot. 2021; DOI: 10.1097/jpo.0000000000000372 29119860

[51] Frossard L, Berg D, Merlo G, Quincey T, Burkett B. Cost comparison of socket-suspended and bone-anchored transfemoral prostheses. J Prosthet Orthot. 2017; 29(4):150–160. DOI: 10.1097/jpo.0000000000000142

[52] Frossard LA, Merlo G, Burkett B, Quincey T, Berg D. Cost-effectiveness of bone-anchored prostheses using osseointegrated fixation: Myth or reality? Prosthet Orthot Int. 2018; 42(3):318–327. DOI: 10.1177/0309364617740239 29119860

[53] Frossard L. Data supporting the 2019 Queensland Artificial Limb Service's science report about innovations of health services and economic evaluation of limb lower bone-anchored prostheses. 2020; Mendeley data. DOI:10.17632/r3b6wdtd8x.1 PMC10443483 37614998

[54] O'Malley SP. Issues facing the Australian Health Technology Assessment Review of medical technology funding. Med J Aust. 2010; 193(1):30–3. DOI: 10.5694/j.1326-5377.2010.tb03737.x 20618111

[55] Bertram MY, Lauer JA, De Joncheere K, Edejer T, Hutubessy R, Kieny MP, et al. Cost-effectiveness thresholds: pros and cons. Bull World Health Organ. 2016; 94(12):925–930. DOI: 10.2471/BLT.15.164418 27994285 PMC5153921

[56] Cohen DJ, Reynolds MR. Interpreting the results of cost-effectiveness studies. J Am Coll Cardiol. 2008; 52(25):2119–26. DOI: 10.1016/j.jacc.2008.09.018 19095128 PMC2716087

[57] Cape J, Beca J, Hoch J. Introduction to cost-effectiveness analysis for clinicians. Univ Tor Med. 2013; 90(3):103–105.

[58] Drummond M, Tarricone R, Torbica A. Incentivizing research into the effectiveness of medical devices. Eur J Health Econ. 2016; 17(9):1055–1058. DOI: 10.1007/s10198-016-0820-3 27492637

[59] Armstrong BK, Gillespie JA, Leeder SR, Rubin GL, Russell LM. Challenges in health and health care for Australia. Med J Aust. 2007; 187(9):485–9. 17979607 10.5694/j.1326-5377.2007.tb01383.x

[60] Gallego G, Casey R, Norman R, Goodall S. Introduction and uptake of new medical technologies in the Australian health care system: a qualitative study. Health Policy. 2011; 102(2-3):152–8. DOI: 10.1016/j.healthpol.2011.04.003 21601934

[61] Frossard L, Debra B. Australian innovations of health services and economic evaluation of bone-anchored prosthesis using osseointegration, in Australian Orthotic Prosthetic Association (AOPA) Congress. 2017; Melbourne, Australia.

[62] Haggstrom EE, Hansson E, Hagberg K. Comparison of prosthetic costs and service between osseointegrated and conventional suspended transfemoral prostheses. Prosthet Orthot Int. 2013; 37(2):152–60. DOI: 10.1177/0309364612454160 22907950

[63] Hansson E, Hagberg K, Cawson M, Brodtkorb TH. Patients with unilateral transfemoral amputation treated with a percutaneous osseointegrated prosthesis: a cost-effectiveness analysis. Bone Joint J. 2018; 100-B(4):527–534. DOI: 10.1302/0301-620X.100B4.BJJ-2017-0968.R1 29629586 PMC6503759

[64] Brodtkorb TH, Henriksson M, Johannesen-Munk K, Thidell F. Cost-effectiveness of C-leg compared with non-microprocessor-controlled knees: a modeling approach. Arch Phys Med Rehabil. 2008; 89(1):24–30. DOI: 10.1016/j.apmr.2007.07.049 18164326

[65] Gerzeli S, Torbica A, Fattore G. Cost utility analysis of knee prosthesis with complete microprocessor control (C-leg) compared with mechanical technology in trans-femoral amputees. Eur J Health Econ. 2009; 10(1):47–55. DOI: 10.1007/s10198-008-0102-9 18379831

[66] Cutti AG, Lettieri E, Del Maestro M, Radaelli G, Luchetti M, Verni G, et al. Stratified cost-utility analysis of C-Leg versus mechanical knees: Findings from an Italian sample of transfemoral amputees. Prosthet Orthot Int. 2017; 41(3):227–236. DOI: 10.1177/0309364616637955 27025244

[67] Chen C, Hanson M, Chaturvedi R, Mattke S, Hillestad R, Liu HH. Economic benefits of microprocessor controlled prosthetic knees: A modeling study. J Prosthet Orthot. 2019; 31(1S) 10.1186/s12984-018-0405-8 PMC6157253 30255802

[68] Normann E, Olsson A, Brodtkorb TH. Modular socket system versus traditionally laminated socket: a cost analysis. Prosthet Orthot Int. 2011; 35(1):76–80. DOI: 10.1177/0309364610392812 21515892

[69] Cutti AG, Lettieri E, Verni G. Health technology assessment as theoretical framework to assess lower-limb prosthetics—issues and opportunities from an international perspective. J Prosthet Orthot. 2019; 31(1S):55–73. DOI: 10.1097/jpo.0000000000000235

[70] Gordon R, Magee C, Frazer A, Evans C, McCosker K. An interim prosthesis program for lower limb amputees: comparison of public and private models of service. Prosthet Orthot Int. 2010; 34(2):175–83. DOI: 10.3109/03093640903510980 20184499

[71] Datta D, Harris I, Heller B, Howitt J, Martin R. Gait, cost and time implications for changing from PTB to ICEX sockets. Prosthet Orthot Int. 2004; 28(2):115–20. DOI: 10.1080/03093640408726696 15382805

[72] Highsmith MJ, Kahle JT, Wernke MM, Carey SL, Miro RM, Lura DJ, et al. Effects of the Genium knee system on functional level, stair ambulation, perceptive and economic outcomes in transfemoral amputees. Technol Innov. 2016; 18(2-3):139–150. DOI: 10.21300/18.2-3.2016.139 27917268 PMC5134931

[73] Seelen HA, Hemmen B, Schmeets AJ, Ament AJ, Evers SM. Costs and consequences of a prosthesis with an electronically stance and swing phase controlled knee joint. Technol Disabil. 2009; 21(1,2): 25–34. DOI: 10.3233/TAD-2009-0269

[74] Selles RW, Janssens PJ, Jongenengel CD, Bussmann JB. A randomized controlled trial comparing functional outcome and cost efficiency of a total surface-bearing socket versus a conventional patellar tendon-bearing socket in transtibial amputees. Arch Phys Med Rehabil. 2005; 86(1):154–61; quiz 180. DOI: 10.1016/j.apmr.2004.03.036 15641007

[75] Husereau D, Drummond M, Petrou S, Carswell C, Moher D, Greenberg D, et al. Consolidated health economic evaluation reporting standards (CHEERS)–explanation and elaboration: a report of the ISPOR Health Economic Evaluation Publication Guidelines Good Reporting Practices Task Force. Value Health. 2013; 16(2):231–50. DOI: 10.1016/j.jval.2013.02.002 23538175

[76] Van Mastrigt GA, Hiligsmann M, Arts JJ, Broos PH, Kleijnen J, Evers SM, et al. How to prepare a systematic review of economic evaluations for informing evidence-based healthcare decisions: a five-step approach (part 1/3). Expert Rev Pharmacoecon Outcomes Res. 2016; 16(6):689–704. DOI: 10.1080/14737167.2016.1246960 27805469

[77] Gerkens S, Crott R, Cleemput I, Thissen JP, Closon MC, Horsmans Y, et al. Comparison of three instruments assessing the quality of economic evaluations: a practical exercise on economic evaluations of the surgical treatment of obesity. Int J Technol Assess Health Care. 2008; 24(3):318–25. DOI: 10.1017/s0266462308080422 18601800

[78] Edwards DS, Phillip RD, Bosanquet N, Bull AM, Clasper JC. What is the magnitude and long-term economic cost of care of the British Military Afghanistan Amputee Cohort? Clin Orthop Relat Res. 2015; 473(9):2848–55. DOI: 10.1007/s11999-015-4250-9 26028596 PMC4523526

[79] Dobson A, El-Gamil A, Shimer M, DaVanzo JE. Economic value of prosthetic services among medicare beneficiaries: A claims-based retrospective cohort study, 2011-2014. J Neuroeng Rehabil. 2018;5;15(Suppl 1):55. DOI: 10.1186/s12984-018-0406-7 30255806 PMC6157184

[80] Sonnenberg FA, Beck JR. Markov models in medical decision making: a practical guide. Med Decis Making. 1993; 13(4):322–38. DOI: 10.1177/0272989x9301300409 8246705

[81] Blough DK, Hubbard S, McFarland LV, Smith DG, Gambel JM, Reiber GE. Prosthetic cost projections for servicemembers with major limb loss from Vietnam and OIF/OEF. J Rehabil Res Dev. 2010; 47(4):387–402. DOI: 10.1682/jrrd.2009.04.0037 20803406

[82] Briggs A, Sculpher M, Buxton M. Uncertainty in the economic evaluation of health care technologies: the role of sensitivity analysis. Health Econ. 1994; 3(2):95–104. DOI: 10.1002/hec.4730030206 8044216

[83] Basu, A. and M.L. Maciejewski, Choosing a Time Horizon in Cost and Cost-effectiveness Analyses. JAMA, 2019. 321(11): p. 1096. DOI: 10.1001/jama.2019.1153. 30789668

[84] Wijnen BF, Van Mastrigt GA, Redekop WK, Majoie HJ, De Kinderen RJ, Evers SM. How to prepare a systematic review of economic evaluations for informing evidence-based healthcare decisions: data extraction, risk of bias, and transferability (part 3/3). Expert Rev Pharmacoecon Outcomes Res. 2016; 16(6): p. 723–732. DOI: 10.1080/14737167.2016.1246961 27762640

[85] Thielen FW, Van Mastrigt GA, Burgers LT, Bramer WM, Majoie HJ, Evers SM, et al. How to prepare a systematic review of economic evaluations for clinical practice guidelines: database selection and search strategy development (part 2/3). Expert Rev Pharmacoecon Outcomes Res, 2016. 16(6): p. 705–721. DOI: 10.1080/14737167.2016.1246962 27805466

[86] Frossard L, Stevenson N, Sullivan J, Uden M, Pearcy M. Categorization of activities of daily living of lower limb amputees during short-term use of a portable kinetic recording system: A preliminary study. J Prosthet Orthot. 2011; 23(1):2–11. DOI: 10.1097/JPO.0b013e318207914c

